# Sorafenib versus Transarterial chemoembolization for advanced-stage hepatocellular carcinoma: a cost-effectiveness analysis

**DOI:** 10.1186/s12885-018-4308-7

**Published:** 2018-04-05

**Authors:** Shuling Chen, Zhenwei Peng, Mengchao Wei, Weifeng Liu, Zihao Dai, Haibo Wang, Jie Mei, Mingfong Cheong, Hanmei Zhang, Ming Kuang

**Affiliations:** 1 Division of Interventional Ultrasound, The First Affiliated Hospital of Sun Yat–sen University, Guangzhou, 510080 China; 2 Department of Oncology, The First Affiliated Hospital of Sun Yat–sen University, Guangzhou, 510080 China; 3 Department of Liver Surgery, The First Affiliated Hospital of Sun Yat–sen University, Guangzhou, 510080 China; 4 Department of Anesthesiology, The First Affiliated Hospital of Sun Yat–sen University, Guangzhou, 510080 China; 5 Clinical Research Unit, The First Affiliated Hospital of Sun Yat–sen University, Guangzhou, 510080 China; 6 Zhongshan School of Medicine, Sun Yat-sen University, Guangzhou, 510080 China

**Keywords:** Sorafenib, Transarterial chemoembolization, Advanced-stage hepatocellular carcinoma, Cost-effectiveness analysis, Markov model

## Abstract

**Background:**

Sorafenib and transarterial chemoembolization (TACE) might both provide survival benefit for advanced hepatocellular carcinoma (HCC). Adopting either as a first-line therapy carries major cost and resource implications. We aimed to estimate the cost-effectiveness of sorafenib and TACE in advanced HCC.

**Methods:**

A Markov model was constructed in a hypothetical cohort of patients aged 60 years with advanced HCC and Child-Pugh A/B cirrhosis over a 2-year time frame. Three strategies (full or dose-adjusted sorafenib and TACE) were compared in two cost settings: China and the USA. Transition probabilities, utility and costs were extracted from systematic review of 27 articles. Sensitivity analysis and Monte Carlo analysis were conducted.

**Results:**

Full and dose-adjusted sorafenib respectively produced 0.435 and 0.482 quality-adjusted life years (QALYs) while TACE produced 0.375 QALYs. The incremental cost-effectiveness ratio (ICER) of full-dose sorafenib versus TACE was $101,028.83/QALY in China whereas full-dose sorafenib is a dominant strategy (ICER of -$1,014,507.20/ QALY) compared with TACE in the USA. Compared to full-dose sorafenib, dose-adjusted sorafenib was the dominant strategy with the negative ICERs in both China (−$132,238.94/QALY) and the USA (−$230,058.09/QALY). However, dose-adjusted sorafenib is not available currently, so full-dose sorafenib should be compared with TACE. As the acceptability curves shown, full-dose sorafenib was the optimal strategy at the accepted thresholds of WTP in these two countries. Specifically, full-dose sorafenib was the cost-effective treatment compared with TACE if a WTP was set above $21,670 in the USA, whereas in China, TACE could be more favorable than full-dose sorafenib if a WTP was set below $10,473.

**Conclusions:**

Dose-adjusted sorafenib may be cost-effective compared to full-dose sorafenib or TACE for advanced HCC patients. However, when confining the comparisons between full-dose sorafenib and TACE, full-dose sorafenib was cost-effective for these patients, under the accepted thresholds of WTP.

**Electronic supplementary material:**

The online version of this article (10.1186/s12885-018-4308-7) contains supplementary material, which is available to authorized users.

## Background

Hepatocellular carcinoma (HCC) is the fifth most common cancer and the third most common cancer-related cause of death, and carries a substantial healthcare spending burden worldwide [1]. Despite recent improvements in surveillance programs, a considerable proportion of patients have vascular invasion or extrahepatic metastasis (advanced stage) at time of diagnosis [2, 3]. Sorafenib, an oral multi-kinase inhibitor, is the standard systemic therapy in the treatment of advanced HCC, based on two multicenter randomized controlled trials (RCTs), which demonstrate improved overall survival of full-dose sorafenib compared with best supportive care (BSC) [4, 5]. In routine clinical practice, sorafenib is recommended to be administered as 800 mg daily, based on the RCT data. However, a substantial portion of patients receiving sorafenib require dose adjustment due to the relatively high rate of adverse effects during the treatment period in the real clinical setting. Moreover, the high cost of full-dose sorafenib is a heavy financial burden for patients with advanced HCC. Recent studies have suggested that a dose-adjusted sorafenib regimen might achieve a better efficacy-safety balance [6–8]. Nevertheless, dose-adjusted sorafenib has not been recommended by the current guidelines due to lack of enough robust data.

Transarterial chemoembolization (TACE) is the standard treatment for patients with intermediate-stage HCC [9]. However, intrahepatic tumor control with TACE might be reasonable and beneficial for advanced HCC considering the fact that more than two-thirds of patients with advanced HCC die of liver failure or intrahepatic tumor progression [10–12]. Some studies have already reported the potential benefits of TACE for patients in this stage [12–21], even after sorafenib was universally established as the first-line treatment. Although several comparative studies tried to compare efficacy between TACE and sorafenib in advanced HCC [14, 15, 22, 23], results were controversial and no RCT has been performed to address this question. It remains unknown how TACE compares with sorafenib for treatment of advanced HCC. Particularly, dose-adjusted regimen is not available when sorafenib is first administered, so full-dose sorafenib will be compared with TACE. Moreover, there may be a significant difference in costs among these three treatments, which may significantly impact the treatment selection due to practical considerations.

Until now, no cost-effectiveness (CE) analysis has been performed on this issue. The Markov model would be a proper solution for this issue. By dividing a disease into distinct states and assigning transitions probabilities for movement between those states, then attaching estimates of costs and health outcomes to the states and running the model over many cycles, the model is able to estimate the long-term costs and outcomes associated with that disease and the related healthcare interventions [24]. The advantage of Markov model by taking into accounts both costs and outcomes over a period of time makes it particularly suited to evaluate the cost-effectiveness of strategies in the treatment of chronic disease. For example, Markov model is widely adopted in the evaluation of disease screening or treatments around the world [25–27]. Therefore, we aimed to construct a Markov model to evaluate and compare the CE of full-dose, dose-adjusted regimens of sorafenib and TACE for advanced HCC, in order to provide useful information for patients and healthcare providers in the current era of limited health resources.

## Methods

### Model construction

**Fig. 1:**
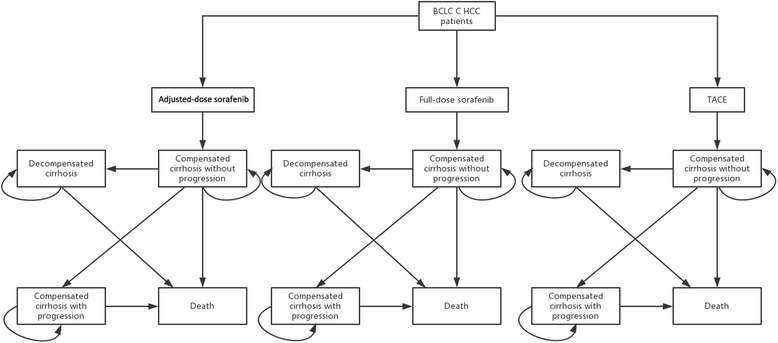
Flow diagram of Markov cohort model. Each pane represents a state of health. Straight lines with arrows indicate transition from one state to another one while circular arrows mean that some patients may stay at the same state for more than one cycle

A Markov model (Fig. 1) was designed to estimate the effects and costs of TACE and sorafenib in a hypothetical cohort of adult patients with advanced HCC (Barcelona Clinic Liver Cancer (BCLC) stage C, patients with Child-Pugh class A/B liver function with vascular invasion or extrahepatic spread or symptoms (Eastern Cooperative Oncology Group Performance Status 1–2)) over a 2-year time frame. The outcome measures were life year gain (LYG), quality-adjusted life expectancy (QALY) and incremental cost-effectiveness ratio (ICER). The model consisted of three decision strategies including full-dose sorafenib, dose-adjusted sorafenib and TACE. Since liver function and tumor burden are the two main factors associated with survival and costs [28, 29], four health states were derived such as compensated cirrhosis (defined as Child-Pugh A/B cirrhosis) with or without progression, decompensated cirrhosis (Child-Pugh C cirrhosis) and death. Tumor progression was defined according to the modified Response Evaluation Criteria of Solid Tumor (mRECIST) [30]. Tumor progression was not considered in the state of decompensated cirrhosis because decompensation was the major life-threatening factor, and the utility and total costs of patients in this state with or without progressive HCC would not be much different [31]. The treatment response was evaluated by mRECIST in compensated cirrhotic patients after administering sorafenib or TACE for HCC. Patients with progressive HCC or decompensated cirrhosis were assumed to receive no further active treatments. Considering the poor prognosis of advanced HCC, the cycle time was set to be one month. A 2-year follow-up duration was assumed because the median survivals of advanced HCC patients were generally less than 10 months. In each cycle, patients in one health state might be transitioned to another, or could occupy the same state, according to transition probabilities. Each health state had its corresponding costs and utilities. Monthly transition probabilities were estimated from the original data using the declining exponential approximation of life expectancy (DEALE) equation [32]. The TreeAge–Pro–2011 software (TreeAge Software Inc., Williamstown, MA, USA) was applied to create a Markov model.

### Literature review

Transition probabilities, utilities and costs (Tables 1, 2 and 3) were derived from published articles identified through PubMed and Cochrane Library database with the latest search performed on July 15, 2016. The medical subject heading (MESH) or their free text variants such as “Carcinoma, Hepatocellular”, “BCLC C” or “advanced HCC”, “sorafenib”, “Transarterial chemoembolization” or “TACE”, and “costs and cost analysis” were applied. Reference lists of the included studies were hand-searched to identify further relevant articles. More details of literature review were in Additional file 1. The details of references used to extract various probabilities were reported in Additional file 2: Table S1, Additional file 3: Table S2, Additional file 4: Table S3, Additional file 5: Table S4, Additional file 6: Table S5, Additional file 7: Table S6, Additional file 8: Table S7, Additional file 9: Table S8, Additional file 10: Table S9.

**Table 1:** Base Case Value and Range of Transition Probabilities

Variables	Base-case value	Range
Background mortality	Age-specific^31^	–
Median survival of decompensated cirrhotic patients (months)^36,37, ßŒ^		1.80–6.00
Derived monthly mortality of decompensated cirrhotic patients (%)	19.00	10.91–31.96
Rates in patients with compensated cirrhosis and advanced HCC		
Median survival after TACE (months)^16–19, 38–40, ßŒ^		4.70–9.50
Derived monthly mortality rate after TACE (%)	11.50	7.04–13.71
Time to progression after TACE (months) ^41,42, ßŒ^	3.20	1.90–3.20
Derived monthly tumor progression rate after TACE (%)	19.46	19.46–30.57
Monthly decompensation rate after TACE (%)^a^	2.40	1.30–3.50
Median survival after taking sorafenib in full dose (months)^4,39,42, 44,45,46, ßŒ^	9.70	3.30–9.70
Derived monthly mortality rate after taking sorafenib in full dose (%)	6.90	6.90–18.95
Time to progression afrer taking sorafenib in full dose (months) ^4,42,44,45, ßŒ^	4.90	2.70–4.90
Derived monthly tumor progression rate after taking sorafenib in full dose (%)	13.19	11.84–22.64
Median survival after taking sorafenib in adjusted dose (months) ^6–8, ßŒ^		7.80–15.0
Derived montly mortality rate after taking sorafenib in adjusted dose (%)	6.50	4.52–8.50
Time to progression after taking sorafenib in adjusted dose (months) ^8, ßŒ^	9.20	6.40–12.0
Derived monthly tumor progression rate after taking sorafenib in adjusted dose (%)	7.26	5.61–10.26
Monthly decompensation rate after taking sorafenib in full dose or adjusted dose^b^	0.90	0.60–1.40
Rates in patients with compensated cirrhosis and progressive HCC		
Median survival after TACE (months) ^36,37,43, ßŒ^		1.80–6.90
Derived monthly mortality rate after TACE (%)	11.20	9.43–31.96
Median survival after taking sorafenib in full dose or adjusted dose (months) ^46,47, ßŒ^	4.60	2.70–6.60
Derived monthly mortality rate after taking sorafenib in full dose or adjusted dose (%)	#13.99	9.97–22.64

#In this model, we assumed that the mortalities of progressive HCC patients and the decompensation rate after full-dose or dose-adjusted sorafenib treatment were the same. More details could be seen in the Materials and Methods

ß refer to additional file Tables for detailed reference lists and original probabilities

Œ all probabilities were transformed into monthly rate. Detailed transformation methods were reported in the notes of corresponding additional file Tables

^a^the data were obtained from the large HCC database of South China (http://hcc.medidata.cn/)

**Table 2:** Base-Case Value and Sensitivity Range for Costs

Variables	Base case value	Range
*Costs in China($)*		
Monthly cost of sorafenib and TACE		
Full-dose Sorafenib		
The first 3 months without supportive policy^48^	7600.38	3800.19–15,200.76
3 months after^48^	15.06	7.53–30.11
Dose-adjusted Sorafenib		
The first 3 months without supportive policy^48^	3807.72	1903.86–7615.43
3 months after^48^	15.06	8.03–32.11
TACE per session	3347.97	1673.99.23–6695.95
Monthly cost of progressive HCC^#^	247.43	123.71–494.86
Monthly cost of decompensated cirrhosis^a,b^	1131.82	565.91–2263.65
Monthly cost of compensated cirrhosis^a,b^	344.14	172.07–688.29
*Costs in the USA($)*		
Monthly cost of sorafenib and TACE		
Full-dose Sorafenib		
The first 3 months without supportive policy^50^	4592.4	2296.2–9184.8
3 months after^50^	1.2	0.60–2.40
Dose-adjusted Sorafenib		
The first 3 months without supportive policy^50^	2296.2	1148.10–4592.40
3 months after^50^	1.2	0.60–2.40
TACE per session^49^	25,961.00	12,980.50–51,922.0
Monthly cost of progressive HCC^51#^	8072	4036.00–16,144.00
Monthly cost of decompensated cirrhosis^49#^	1519.0	759.50–3038.00
Monthly cost of compensated cirrhosis^49#^	61.0	30.0–122.00

^a^The data were obtained from the large HCC database of South China (http://hcc.medidata.cn/)

^b^The total monthly cost of drugs and procedures (that include treatments for HCC, cirrhosis and adverse events derived from associated drugs and procedures), inpatient and outpatient visits, laboratory testing and imaging examination

**Table 3:** Base Case Value and Range of Utilities Extracted from Literatures

Variables	Base case value	Range
Compensated cirrhosis without progressive HCC^52^	0.76	0.76–0.80
Compensated cirrhosis with progressive HCC^52^	0.68	0.60–0.68
Decompensated cirrhosis^53^	0.57	0.46–0.68

### Parameter estimation

Double arcsine transformations were performed on the extracted transition probabilities before pooling them for variances stabilisation because the inverse variance weight in the pooling is suboptimal when low prevalence rates are involved and the transformed probabilities are weighted very slightly towards 50%, making it feasible to include studies with prevalence rates of zero [33]. The Wilson score method was also used to calculate the 95% confidence intervals (CIs) of these probabilities because of the values below zero produced by the asymptotic method [34]. After obtaining the above estimates, STATA software (Stata Corp., College Station, TX, USA) was used to pool the data using the random-effect model. SAS 9.2 (SAS Institute Inc., Cary, NC, USA) was used to apply the Wilson score method and calculate the 95% CIs.

### Summary of transition probabilities

#### Common transition probabilities

The common probability for these three therapies was the mortality of the general population [35] and of decompensated cirrhotic patients. Advanced HCC patients with decompensated cirrhosis were regarded as candidates with terminal stage HCC without chances to receive further effective interventions, so the natural mortality of terminal-stage patients was adopted [36, 37].

#### Transition probabilities in TACE arm

Studies on the risk of transitioning from compensated to decompensated state for cirrhotic patients with advanced HCC were unavailable. The monthly decompensated rate for compensated cirrhotic patients with advanced HCC but without tumor progression after TACE was 2.40%, extracting from the large HCC database of South China (http://hcc.medidata.cn/). This is a constantly updated database comprising primary and recurrent HCC patients treated in a tertiary medical center. There are 1694 patients with follow-up data by May 2017. Some of the data in our study are extracted from the cohort of primary advanced-stage HCC patients treated with sorafenib or TACE in this database. The monthly mortality of patients without progression after TACE was estimated as 11.5% after pooling the data from nine included studies[16–19, 38–40]. The progression rate after TACE was transformed from the time to progression (TTP) of 3.2 months reported by Zhou et al. [41, 42]. There was no study specifically reporting the mortality rate of progressive disease after TACE, so we assumed that the mortality was comparable to the median of natural mortality of advanced and terminal stage HCC since there was no further treatment for post-progression patients in this model [36, 37, 43].

#### Transition probabilities in full-dose/adjusted-dose Sorafenib arm

Studies on the risk of transitioning from compensated to decompensated state for cirrhotic patients with advanced HCC were unavailable. The monthly decompensated rate for compensated cirrhotic patients with advanced HCC but without tumor progression after sorafenib treatment was 0.90%, extracting from the large HCC database of South China (http://hcc.medidata.cn/). For the full-dose sorafenib cohort without progression, the median survival of 9.7 months and the TTP of 4.9 months were extracted from a sub-analysis of the SHARP trial [42]. Other studies have applied the supplemental survival and progression data for analysis [4, 39, 44–46]. For the monthly mortality of patients without progression after taking adjusted-dose sorafenib, 6.5% was resulted after pooling all the data from included studies, [6–8]. The TTP of 9.2 months was derived from the SOFIA study in Italy and its 95% confidence interval (CI) was used to perform a sensitivity analysis [8]. Median post-progression survival was consistently reported as 4.6 months in two studies [46, 47]. We assumed that the survival of patients after sorafenib failure in both arms shared the same scenario.

### Costs and utilities

This study was conducted from the perspective of a healthcare system; therefore, only direct medical costs were included. Since China and the USA present with different medical charging systems with different costs for procedures, administering the treatment (that includes doctor visits) and follow-up monitoring, we performed two cost scenarios: in China and the USA. Monthly costs were estimated with the frequency and unit cost of drugs and procedures (that include treatments for HCC, cirrhosis and adverse events derived from associated drugs and procedures), inpatient and outpatient visits, laboratory testing and imaging examination, and all were converted to U.S. dollars in 2016. The costs in China were extracted from a previously published Chinese study [48], supplemented by the data in the large HCC database of South China (http://hcc.medidata.cn/). The median cost of one session of TACE was $3347.97. The market price for a package of sorafenib is $3792.66 in China. We also considered the cost reduction due to the sorafenib assistance program for patients without progression until 3 months in China during the analysis. In this program, patients need to pay the drug cost for the initial 3 months, and then are free of charge until the end of treatment. The cost estimates in the USA were obtained from cost-relative studies specific for the USA. The median cost of one session of TACE was $25,961 [49] and the market price for a tablet of sorafenib is $38.27 [50]. Other costs were also available in the literatures [49, 51]. The base case estimates and sensitivity ranges of utilities were extracted from the literatures [52, 53]. A discount rate was set at 3% yearly for both costs and utilities to inflate costs to 2016 dollars.

### Cost-effectiveness analysis

With the above parameters input into the model, effectiveness measures of LYG and QALY and CE measure of ICER were estimated. The ICER was calculated using the difference in costs divided by the corresponding difference in QALY. The relative cost-effectiveness among the three strategies were compared with one another in order of increasing effectiveness [54]. Subsequently, one-way sensitivity analysis was performed to evaluate the effect of each parameter preset in our model. To reduce the ambiguity of the mathematical uncertainty of ICER, a new assessment criterion, net monetary benefits (NMB), was used. The formula of NMB combines cost, effectiveness and willingness to pay (WTP). The strategy with the higher NMB is more cost-effective given the fixed WTP value. Results were presented in the form of a tornado diagram and the corresponding cutoffs were determined. For probabilities and utilities, we changed the value of each parameter over the range extracted from the included studies. For costs, considering the lack of reported range data, a wide range of 50%–200% on the base case value was applied as Lim et al. [49] described. Furthermore, Monte Carlo probabilistic sensitivity analysis was performed to estimate the total impact of parameter uncertainties on the model results with 10,000 simulations. Findings were presented on a CE acceptability curve and a CE plane. A gamma distribution was employed for cost estimates and a beta distribution for efficacy estimates.

An external CE threshold, that is, the largest sum of money you are willing to pay for gaining one QALY was utilized to compare with ICER to decide whether one strategy is cost-effective. For the USA, the commonly cited threshold of $50,000/QALY was used [49, 55, 56]. For China, we adopted the threshold of $24,840.27 which is the 3 times per capita GDP of China according to the WHO guidelines for CE analysis [48, 57].

## Results

### Base case analysis

Tables 4 and 5 summarize the results of base case analyses. For LYG, the dose-adjusted -sorafenib strategy provided an average of 7.898 months while TACE and full-dose sorafenib strategies offered 6.357 months and 7.236 months, respectively. Regarding QALY, full-dose sorafenib and dose-adjusted sorafenib treatments produced relatively better results (0.435, 0.482) than TACE (0.375). In China, full-dose sorafenib brings 0.060 of a QALY per person at a cost of $ 6061.73 per person when compared to TACE, which yields an ICER of $101,028.83 per QALY whereas in the USA, full-dose sorafenib is a dominant strategy (ICER of -$1,014,507.20/ QALY) compared with TACE. Compared to full-dose sorafenib, dose-adjusted sorafenib was the dominant strategy with the negative ICERs in both China (−$132,238.94/QALY) and the USA (−$230,058.09/QALY).

**Table 4:** Base Case Values of Cost-Effectiveness Analysis of Three Strategies in China and the USA

Parameters	China			USA		
	TACE	Full-dose Sorafenib	Dose-adjusted Sorafenib	TACE	Full-dose Sorafenib	Dose-adjusted Sorafenib
LYG (months)	6.357	7.236	7.898	6.357	7.236	7.898
QALYs (years)	0.375	0.435	0.482	0.375	0.435	0.482
Lifetime cost ($)	10,642.22	16,703.95	10,488.72	95,061.13	34,190.70	23,377.97.54

**Table 5:** Incremental Cost-Effectiveness Ratios Comparing Three Strategies in China and the USA

	China		USA	
	Full-dose sorafenib vs TACE	Dose-adjusted sorafenib vs full-dose sorafenib	Full-dose sorafenib vs TACE	Dose-adjusted sorafenib vs full-dose sorafenib
Incremental cost per person ($)	6061.73	-6215.23	−60,870.43	−10,812.73
Incremental QALYs per person (years)	0.060	0.047	0.060	0.047
Incremental cost per QALY (ICER, $)	101,028.83	−132,238.94	−1,014,507.20	−230,058.09

### One-way sensitivity analysis

**Fig. 2:**
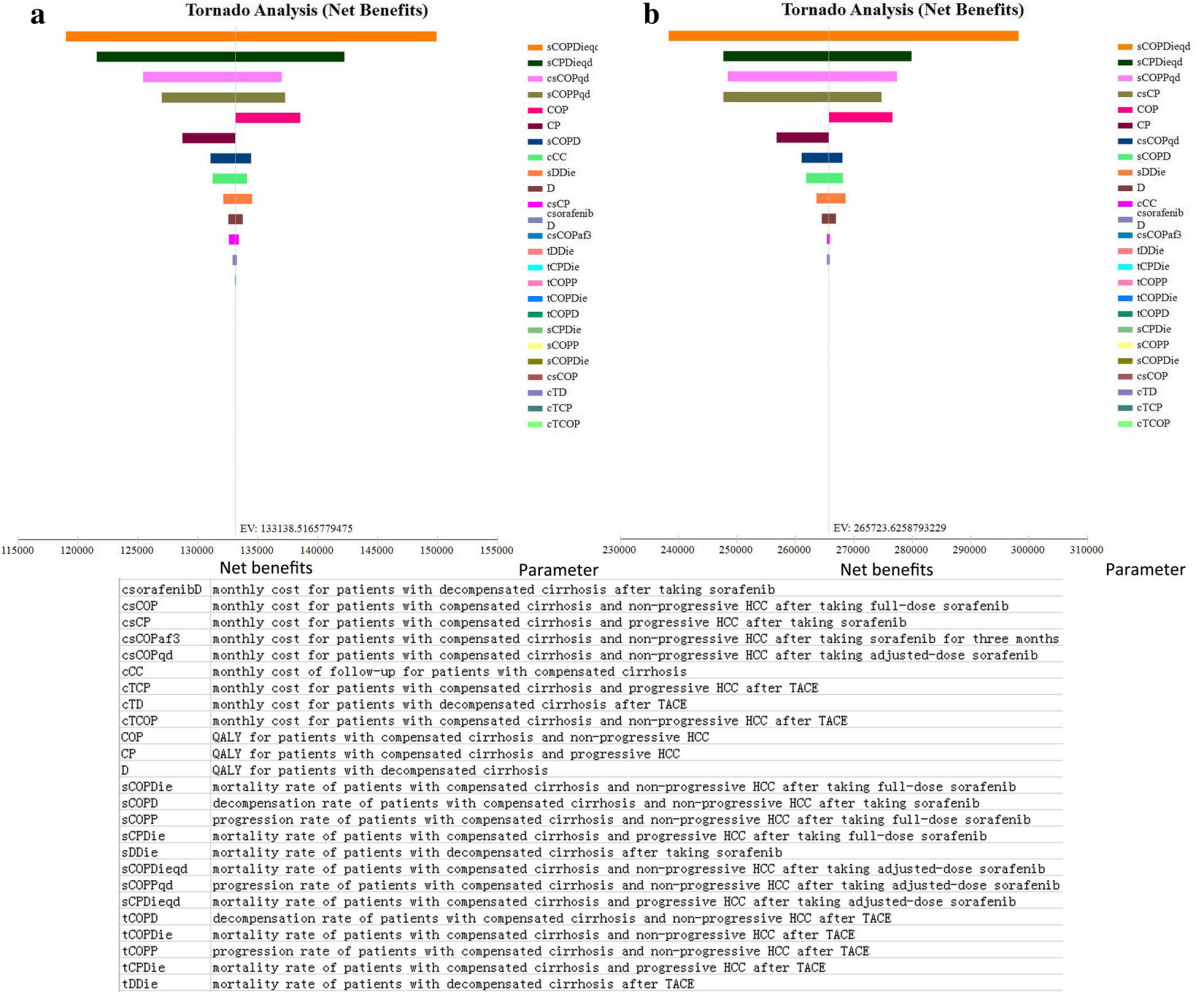
Tornado diagrams of one-way sensitivity analyses for China (**a**) and the USA (**b**). All transition probabilities and costs defined in this model were analyzed. The length of colored bar for each factor represents the extent of its effect on NMB. A wider bar of the corresponding variable indicates the larger potential effect on NMB

Fig. 2 demonstrates the tornado diagrams of all the parameters for China and the USA. The mortalities of compensated cirrhotic patients without and with progression taking dose-adjusted sorafenib lay in the top three sensitive parameters in both countries, reflecting that the treatment efficacy was a vital factor when considering the sorafenib strategy. For both China and the USA, the NMB of dose-adjusted sorafenib treatment was always larger than those of full-dose sorafenib and TACE regardless of the monthly mortality of compensated cirrhotic patients with progression taking dose-adjusted sorafenib (Additional file 11: Figure 1A, B), suggesting dose-adjusted sorafenib was the dominant strategy over the range we tested. Besides, the cost of sorafenib was the third sensitive parameter in China. If the expenditure of full-dose sorafenib treatment increased and reached the cut-off value of $13,973.90, TACE could become more competitive (Additional file 12: Figure 2A). In the USA, TACE was apparently not a good treatment under the given scenarios reagardless of the cost of full-dose sorafenib treatment (Additional file 12: Figure 2B).

### Two-way sensitivity analysis

**Fig. 3:**
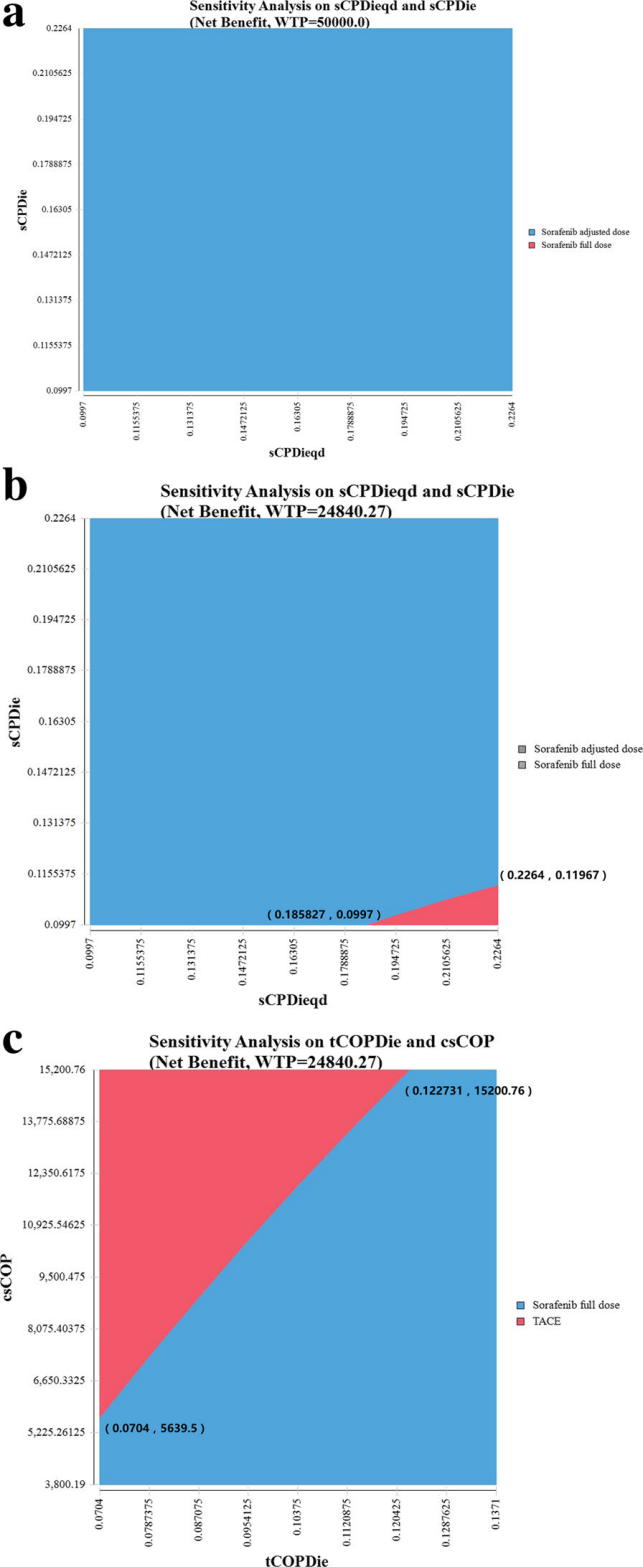
**a** Two-way sensitivity analysis of sCPDie and sCPDieqd for the NMB in the USA. **b** Two-way sensitivity analysis of sCPDie and sCPDieqd for the NMB in China. **c** Two-way sensitivity analysis of tCOPDie and csCOP for the NMB in China. sCPDie: the mortality of compensated cirrhotic patients with progression taking sorafenib in full dose; sCPDieqd: the mortality of compensated cirrhotic patients with progression taking sorafenib in adjusted dose; tCOPDie: the mortality of compensated cirrhotic patients without progression taking sorafenib in full dose; csCOP: the cost of sorafenib for compensated cirrhotic patients without progression taking sorafenib in full dose

The top three sensitive parameters were further included in the two-way sensitivity analysis. In the USA scenario, the analysis demonstrated that dose-adjusted sorafenib was always the cost-effective treatment no matter how the post-progression survivals of sorafenib treated HCC patients changed (Fig. 3a). In China, most scenarios suggested that dose-adjusted sorafenib mode was more cost-effective. However, when assuming the lowest mortality to patients with progression taking full-dose sorafenib, adjusted-dose regimen remained cost-effective until the monthly mortality reached 18.6% (Fig. 3b). For TACE and full-dose sorafenib therapies, if the drug cost of full-dose sorafenib was kept at $15,200.8, the monthly mortality of advanced HCC patients without progression would need to be kept below 12.3% for TACE to be cost-effective in China (Fig. 3c).

### Monte Carlo analysis

**Fig. 4:**
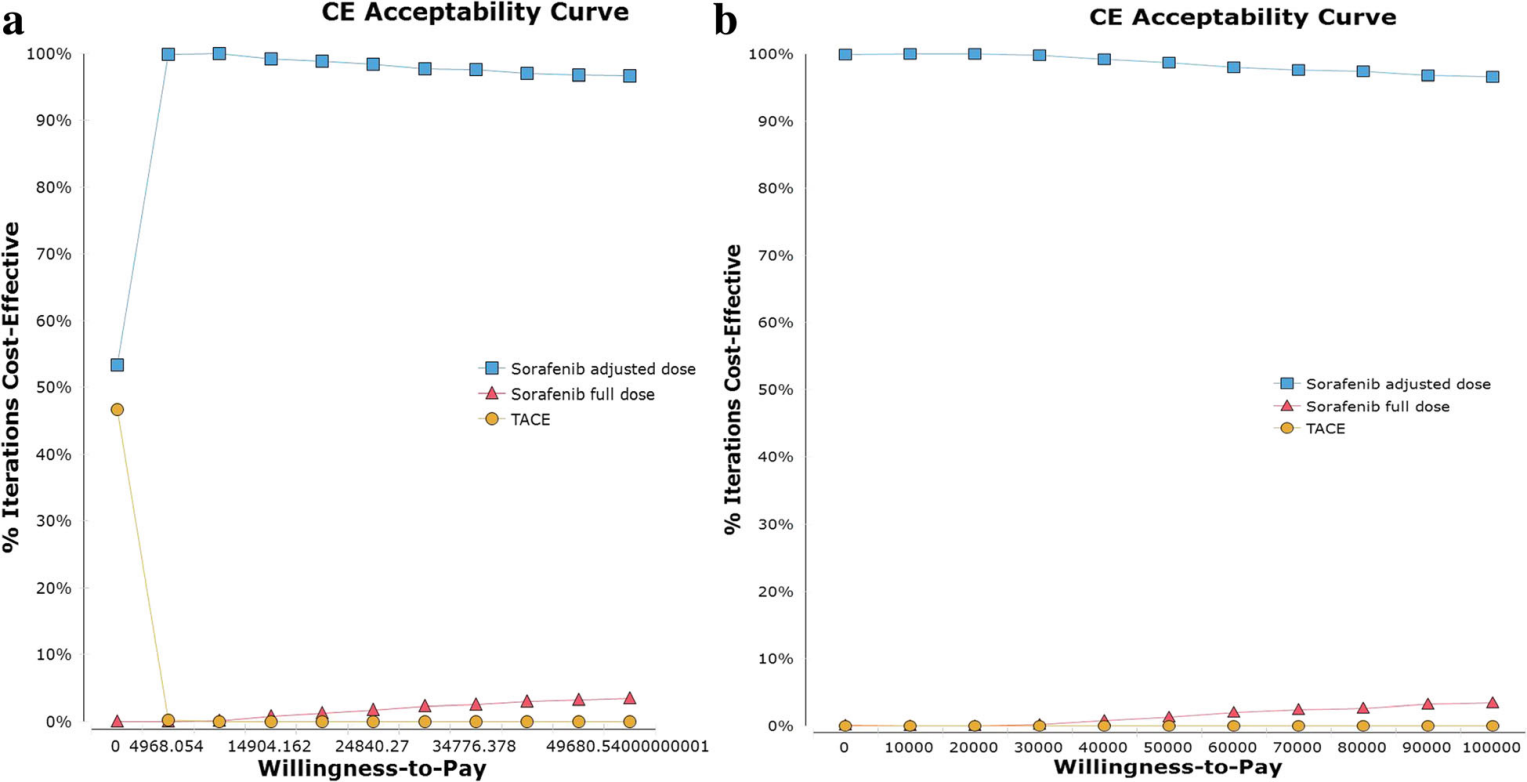
Cost-effectiveness acceptability curve (CEAC) of the three treatment strategies for China (**a**) and the USA (**b**). CEAC represented the uncertainty in cost-effectiveness analysis and provided the reference to the WTP thresholds

The median ICERs of full-dose sorafenib and TACE compared to dose-adjusted sorafenib were − 6623.21(− 25,354.58,12,108.17)and − 377.23(− 477.03,-277.42)in China and 583,742.25 (− 535,297.82,1702,782.31) and − 65,695.39 (− 70,138.02,− 61,252.76) in the USA, respectively. For both China and the USA, the acceptability curve showed that dose-adjusted sorafenib always had a higher probability of being more cost-effective than the other two therapies regardless of WTP (Fig. 4a, b). Moreover, in the USA, TACE could be as cost-effective as full-dose sorafenib if the WTP was below $21,670 while full-dose sorafenib was always cost-effective compared with TACE if the WTP was set above $21,670. Nevertheless, in China, TACE could be more favorable than full-dose sorafenib if a WTP was set below $10,473 (Fig. 4a). Despite the above disparities, the common finding in these two countries was that dose-adjusted sorafenib was the cost-effective strategy at the preset WTP values in China and USA, and when confining the comparisons between full-dose sorafenib and TACE, full-dose sorafenib was cost-effective for these patients.

## Discussion

Our study shows that dose-adjusted sorafenib is cost-effective compared to full-dose sorafenib or TACE for advanced HCC in both China and the USA based on the base case analysis, sensitivity analysis and WTP analysis. To the best of our knowledge, this is the first study to compare the CE of sorafenib with TACE. Previous CE studies have confined the comparison of sorafenib to BSC [48, 50, 55, 58]. However, since BSC is simply a form of palliative treatment without offering improvement in health outcomes, comparing an active treatment like TACE to sorafenib is a better strategy when investigating the current most appropriate therapy for advanced HCC. Moreover, it is also the first study to compare sorafenib in different doses (full-dose and dose-adjusted) with TACE in the treatment of advanced HCC.

Regarding the CE of full-dose sorafenib itself, a QALY of 0.435 gained at a cost of $16,703 in China in our study was similar to that in the previous Chinese study (QALY, 0.45; cost, $19,149) [48], but was not consistent with that in the Italian study (QALY, 0.16; cost, $14,841;) [55]. The poorer efficacy of full-dose sorafenib for Italian patients represented by the much shorter QALY could explain the inconsistency. Meanwhile, for the dose-adjusted sorafenib group, its CE in China in our study were better than that in the Italian study [55], which might be explained by the higher expenditures (Italy: $16,625 vs. China: 10,488) but lower QALY in Italy (Italy: 0.440 vs. China: 0.482).

In terms of the relative CE between therapies, the Italian study and the Chinese study both suggested that full-dose sorafenib was not a cost-effective treatment compared to BSC [48, 55]. However, the Italian study considered sorafenib as cost-effective under the dose-adjusted condition. Similarly in our study, dose-adjusted sorafenib was cost-effective whereas full-dose sorafenib and TACE were not. It might be due to the similar or higher survival benefit provided by dose-adjusted sorafenib at a lower cost. Sorafenib therapy within its licensed dose is effective but costly, thus, it is of great importance to find a way to improve its CE. Half-dose sorafenib with the comparable or even better efficacy and substantial expenditure reduction is clearly a good option. However, the issue of optimal sorafenib dose remained to be further investigated in the future studies.

Concerning the role of TACE in advanced HCC, our study revealed that TACE produced slightly shorter life expectancy and QALY to sorafenib. Although TACE could eradicate viable tumors to some extent, the promotion of proliferation and metastasis of remaining tumors by over-expressed angiogenic and inflammatory factors after TACE restricted the prolongation of the overall survival. Moreover, when taking the cost into consideration, TACE was not the cost-effective treatment under the current WTP set in the USA as shown in the acceptability curve because one session of this procedure ($25,961) costs more than 5.5 times of the monthly cost of full-dose sorafenib ($4592). Interestingly, in China, TACE was cost-effective compared to full-dose sorafenib if the WTP was set below $10,473. The underlying reason behind the difference between China and the USA might be the different medical charging system between these two countries representing by the relative cost of TACE and sorafenib. Unlike the high expense of TACE in the USA [49], the price of TACE including the costs of procedure, hospitalization, specialist visits and various examinations during hospital stay ($3170) in China is even lower than the monthly cost of dose-adjusted sorafenib ($4060). However, under the accepted threshold of WTP in both countries, full-dose usage of sorafenib is still the cost-effective strategy compared with TACE for most patients. Only for some poor patients with the willingness to pay less $10,473 in China, TACE could be the cost-effective treatment.

Data constraints inevitably lead to several limitations within our model. First, there were limited studies that specifically reported TACE and sorafenib outcomes for compensated cirrhotic patients with or without progression. Such a limitation was unfortunately unavoidable in our type of analysis. This limitation obliged us to use the best available data in the literature review. The resulting uncertainties were not significant, which was confirmed by the unchanged results in the probabilistic sensitivity analysis. Second, the utility estimates for compensated cirrhosis with or without progression were extracted from NICE for the treatment of advanced renal carcinoma with sorafenib or BSC as the two previous relevant articles did. This adoption may not be the most rational because utilities might vary between populations with two different diseases. The third limitation concerns the paucity of data on cost estimates for each health state in different countries. It is well known that costs may vary with different regions and treatment plans. Thus, it restricted us to make comparisons among more countries. Despite these, we have performed the analyses on two different cost scenarios including the USA and China, representing the Western and Eastern countries to some extent, and have detected some differences between these two countries. Moreover, we have considered the uncertainties of costs in sensitivity analyses by inputting a wide range of cost values (50%–200% of base-case value). In a sense, our model could be applied in those countries with efficacy and cost data falling within the ranges we have set. Fourth, we assumed that the effectiveness estimations such as LYG and QALY were the same between China and the USA, however, there might be differences in these efficacy results between these two countries. Thus, more specific data for individual countries are required to obtain more accurate results for different countries respectively. Fifth, most of the included studies were retrospective and the analyses based on these retrospective data would inevitably result in selection bias. Sixth, the lack of information on vascular invasion, extrahepatic spread and/or symtoms in TACE versus sorafenib patients was present in our study, which required more detailed characteristics of patients to compare these two treatments in future studies.

## Conclusions

In conclusion, dose-adjusted sorafenib may be cost-effective compared to full-dose sorafenib or TACE for advanced HCC patients. However, dose-adjusted sorafenib is not available currently, so full-dose sorafenib should be compared with TACE. When confining the comparisons between them, full-dose sorafenib was cost-effective compared with TACE for advanced HCC patients, under the accepted thresholds of WTP. Our findings will require further high–quality studies to validate.

## Additional files

Additional file 1: Literature review of this research which includes study strategy, study selection and data extraction. (DOCX 14 kb)

Additional file 2: **Table S1.** References used to derive monthly mortality of advanced HCC patients with compensated cirrhosis without progression after TACE. (DOCX 15 kb)

Additional file 3: **Table S2.** References used to derive monthly tumor progression rate of advanced HCC patients with compensated cirrhosis after TACE. (DOCX 14 kb)

Additional file 4: **Table S3.** References used to derive monthly mortality of advanced HCC patients with compensated cirrhosis without progression taking sorafenib in full dose. (DOCX 13 kb)

Additional file 5: **Table S4.** References used to derive monthly progression rate of advanced HCC patients with compensated cirrhosis taking sorafenib in full dose. (DOCX 13 kb)

Additional file 6: **Table S5.** References used to derive monthly mortality of advanced HCC patients with compensated cirrhosis without progression taking sorafenib in adjusted dose. (DOCX 12 kb)

Additional file 7: **Table S6.** References used to derive monthly progression rate of advanced HCC patients with compensated cirrhosis taking sorafenib in adjusted dose. (DOCX 12 kb)

Additional file 8: **Table S7.** References used to derive monthly mortality of patients with compensated cirrhosis and progressive HCC after TACE. (DOCX 13 kb)

Additional file 9: **Table S8.** References used to derive monthly mortality of patients with compensated cirrhosis and progressive HCC taking sorafenib in full dose or adjusted dose. (DOCX 12 kb)

Additional file 10: **Table S9.** References used to derive monthly mortality of advanced HCC patients with decompensated cirrhosis. (DOCX 12 kb)

Additional file 11: **Figure S1.** One-way sensitivity analysis of sCPDieqd for the NMB in China (A) and the USA (B). The axis of abscissa represented the range of variable showed in Table 1. The Y-axis represented the value of NMB. The strategy with a higher NMB indicates a cost-effective strategy within the preset WTP. As the rate of sCPDieqd increased, the cost-effectiveness of dose-adjusted sorafenib treatment reduced. sCPDieqd: the mortality of compensated cirrhotic patients with progression taking sorafenib in adjusted dose. (TIFF 670 kb)

Additional file 12: **Figure S2.** One-way sensitivity analysis of csCOP for the NMB in China (A) and the USA (B). The axis of abscissa represented the range of variable showed in Table 2. The Y-axis represented the value of NMB. A strategy with a higher NMB indicates a cost-effective strategy within the preset WTP. As the value of csCOP increased, the cost-effectiveness of full-dose sorafenib treatment reduced. csCOP: the cost of sorafenib in compensated cirrhotic patients without progression taking sorafenib in full-dose. (TIFF 694 kb)

## Abbreviations

BSC: Best supportive care
CE: Cost-effectiveness
CI: Confidence interval
DEALE: Declining exponential approximation of life expectancy
HCC: Hepatocellular carcinoma
ICER: Incremental cost-effectiveness ratio
LYG: Life year gain
NMB: Net monetary benefits
QALYs: Quality-adjusted life years
RCTs: Randomized controlled trials
TACE: Transarterial chemoembolization
TTP: Time to progression
WTP: Willingness to pay
