# Supplementary material for: Sorafenib versus Transarterial chemoembolization for advanced-stage hepatocellular carcinoma: a cost-effectiveness analysis
Source: BMC Cancer. 2018 Apr 5;18:392. doi: 10.1186/s12885-018-4308-7 (PMC5887167; doi:10.1186/s12885-018-4308-7)
Supplement: Supplementary file 1 — Literature review of this research which includes study strategy, study selection and data extraction. (DOCX 14 kb) [file 12885_2018_4308_MOESM1_ESM.docx]

**Supplementary Materials and Methods**

**Literature Review**

***Study Strategy***

Literatures were retrieved from two databases of PubMed and Cochrane Library with the latest searching on July 15, 2016. The following search terms were used, as either Medical Subject Headings (MeSH) or text words: hepatocellular carcinoma, liver cancer, primary liver carcinoma, liver cell carcinoma, Barcelona Clinic Liver Cancer stage C, BCLC C, advanced HCC, transarterial chemoembolization, TACE, transarterial embolization, TAE, sorafenib, Nexavar, Raf 1 Kinase Inhibitor II, cost, cost analysis. Boolean operators (AND, OR) were used in succession to create combinations to complete the search. Reference lists from the included studies were hand-searched to identify further relevant literatures. If data subsets were published in more than one article, only the most recent article was included. Citations were limited to those published in English and the search was not restricted by publication year. Two investigators independently searched all the eligible studies and a third individual was consulted when the two evaluators’ opinions differed.

***Study Selection and Data Extraction***

Studies were considered eligible if they met the following criteria: (i) the entire population or subpopulation were adult patients with advanced-stage hepatocellular carcinoma (HCC) who received sorafenib or transarterial chemoembolization (TACE) as their initial treatment; (ii) any of the parameter estimates used in our model was reported, those without the associated probabilities were excluded; (iii) randomized trials, quasi-randomized trials, prospective or retrospective cohort studies were included whereas reviews, letters, case reports, editorials or comments and meeting abstracts were excluded. Subsequently, we extracted all the transition probabilities, utilities and costs from the corresponding included articles. Two investigators independently evaluated all eligible studies and extracted their data. Disagreements were resolved with the discussion with a third investigator.
