# Supplementary material for: Sorafenib versus Transarterial chemoembolization for advanced-stage hepatocellular carcinoma: a cost-effectiveness analysis
Source: BMC Cancer. 2018 Apr 5;18:392. doi: 10.1186/s12885-018-4308-7 (PMC5887167; doi:10.1186/s12885-018-4308-7)
Supplement: Supplementary file 2 — Table S1. References used to derive monthly mortality of advanced HCC patients with compensated cirrhosis without progression after TACE. (DOCX 15 kb) [file 12885_2018_4308_MOESM2_ESM.docx]

**Table S1. References used to derive monthly mortality of advanced HCC patients with compensated cirrhosis without progression after TACE**

| **Reference** | **Author, publication year** | **Centre** | **Sample**  **size** | **Median survival(months)** | **Monthly rate(%)Ψ** |
| --- | --- | --- | --- | --- | --- |
| 14 | Nishikawa H,2012 | Japan | 55 | 6.6 | 9.97 |
| 15 | Pinter M, 2012 | Austria | 34 | 9.2 | 7.26 |
| 16 | Chern MC,2014 | China | 50 | 6.2 | 10.58 |
| 17 | Chung GE, 2011 | Korea | 83 | 5.6 | 11.64 |
| 18 | Georgiades CS, 2005 | USA | 32 | 9.5 | 7.04 |
| 19 | Luo J, 2011 | China | 84 | 7.1 | 9.30 |
| 20 | Kim KM,2008 | Korea | 149 | 4.7 | 13.71 |
| 21 | Lee HS,1996 | Korea | 31 | 5.0 | 12.94 |
| 38 | Liu L, 2014 | China | 188 | 6.5 | 10.11 |

ΨCalculated from the median survival using the following formula:1-(r)^1/time^, r refers to 50% and time refers to median time extracted form literatures.
