# Supplementary material for: Sorafenib versus Transarterial chemoembolization for advanced-stage hepatocellular carcinoma: a cost-effectiveness analysis
Source: BMC Cancer. 2018 Apr 5;18:392. doi: 10.1186/s12885-018-4308-7 (PMC5887167; doi:10.1186/s12885-018-4308-7)
Supplement: Supplementary file 3 — Table S2. References used to derive monthly tumor progression rate of advanced HCC patients with compensated cirrhosis after TACE. (DOCX 14 kb) [file 12885_2018_4308_MOESM3_ESM.docx]

**Table S2. References used to derive monthly tumor progression rate of advanced HCC patients with compensated cirrhosis after TACE**

| **Reference** | **Author, publication year** | **Centre** | **Sample**  **size** | **Time to progression**  **(months)** | **Monthly rate(%)Ψ** |
| --- | --- | --- | --- | --- | --- |
| 39 | Hu H, 2014 | China | 198 | 1.9 | 30.57 |
| 40 | Zhou B, 2016 | China | 76 | 3.2 | 19.46^#^ |

# Selected as the base-case value because it is not only the latest study but also a prospective study among the relevant literatures.

ΨCalculated from the TTP using the following formula:1-(r)^1/time^, r refers to 50% and time refers to TTP extracted form literatures.
