# Supplementary material for: Sorafenib versus Transarterial chemoembolization for advanced-stage hepatocellular carcinoma: a cost-effectiveness analysis
Source: BMC Cancer. 2018 Apr 5;18:392. doi: 10.1186/s12885-018-4308-7 (PMC5887167; doi:10.1186/s12885-018-4308-7)
Supplement: Supplementary file 4 — Table S3. References used to derive monthly mortality of advanced HCC patients with compensated cirrhosis without progression taking sorafenib in full dose. (DOCX 13 kb) [file 12885_2018_4308_MOESM4_ESM.docx]

**Supplementary Table 3. References used to derive monthly mortality of advanced HCC patients with compensated cirrhosis without progression taking sorafenib in full dose**

| **Reference** | **Author, publication year** | **Centre** | **Sample**  **size** | **Median survival**  **(months)** | **Monthly rate(%)Ψ** |
| --- | --- | --- | --- | --- | --- |
| 4 | Cheng AL,2009 | Asia | 118 | 5.6 | 11.64 |
| 15 | Pinter M, 2012 | Austria | 63 | 7.4 | 8.94 |
| 42 | Bruix J,2012 | Europe | 245 | 9.7 | 6.90^#^ |
| 43 | Abou-Alfa GK,2006 | USA | 137 | 9.2 | 7.26 |
| 45 | Cho JY, 2014 | Korea | 35 | 3.3 | 18.95 |

# Selected as the base-case value because it is not only a subanalysis of a RCT study and but also has the largest sample size.

ΨCalculated fromthe median survival using the DEALE method as described above.
