# Supplementary material for: Sorafenib versus Transarterial chemoembolization for advanced-stage hepatocellular carcinoma: a cost-effectiveness analysis
Source: BMC Cancer. 2018 Apr 5;18:392. doi: 10.1186/s12885-018-4308-7 (PMC5887167; doi:10.1186/s12885-018-4308-7)
Supplement: Supplementary file 5 — Table S4. References used to derive monthly progression rate of advanced HCC patients with compensated cirrhosis taking sorafenib in full dose. (DOCX 13 kb) [file 12885_2018_4308_MOESM5_ESM.docx]

**Supplementary Table 4. References used to derive monthly progression rate of advanced HCC patients with compensated cirrhosis taking sorafenib in full dose**

| **Reference** | **Author, publication year** | **Centre** | **Sample**  **size** | **Time to progression**  **(months)** | **Monthly**  **rate(%)Ψ** |
| --- | --- | --- | --- | --- | --- |
| 42 | Bruix J,2012 | Europe | 245 | 4.9 | 13.19^#^ |
| 43 | Abou-Alfa GK,2006 | USA | 137 | 5.5 | 11.84 |
| 44 | Cheng AL,2012 | Asia | 118 | 2.7 | 22.64 |

# Selected as the base-case value because it is not only a subanalysis of a RCT study and but also has the largest sample size.

ΨCalculated from the TTP using the DEALE method as described above.
