# Supplementary material for: Sorafenib versus Transarterial chemoembolization for advanced-stage hepatocellular carcinoma: a cost-effectiveness analysis
Source: BMC Cancer. 2018 Apr 5;18:392. doi: 10.1186/s12885-018-4308-7 (PMC5887167; doi:10.1186/s12885-018-4308-7)
Supplement: Supplementary file 6 — Table S5. References used to derive monthly mortality of advanced HCC patients with compensated cirrhosis without progression taking sorafenib in adjusted dose. (DOCX 12 kb) [file 12885_2018_4308_MOESM6_ESM.docx]

**Supplementary Table 5. References used to derive monthly mortality of advanced HCC patients with compensated cirrhosis without progression taking sorafenib in adjusted dose**

| **Reference** | **Author, publication year** | **Centre** | **Sample size** | **Median survival**  **(months)** | **Monthly**  **rate(%)Ψ** |
| --- | --- | --- | --- | --- | --- |
| 6 | Nishikawa H,2014 | Japan | 88 | 7.8 | 8.50 |
| 7 | Morimoto M,2015 | Japan | 73 | 10.2 | 6.60 |
| 8 | Iavarone M,2011 | Italy | - | 15 | 4.52 |

ΨCalculated from the median survival using the DEALE method as described above.
