# Supplementary material for: Sorafenib versus Transarterial chemoembolization for advanced-stage hepatocellular carcinoma: a cost-effectiveness analysis
Source: BMC Cancer. 2018 Apr 5;18:392. doi: 10.1186/s12885-018-4308-7 (PMC5887167; doi:10.1186/s12885-018-4308-7)
Supplement: Supplementary file 7 — Table S6. References used to derive monthly progression rate of advanced HCC patients with compensated cirrhosis taking sorafenib in adjusted dose. (DOCX 12 kb) [file 12885_2018_4308_MOESM7_ESM.docx]

**Supplementary Table 6. References used to derive monthly progression rate of advanced HCC patients with compensated cirrhosis taking sorafenib in adjusted dose**

| **Reference** | **Author, publication year** | **Centre** | **Sample size** | **Time to progression**  **(months)** | **Monthly**  **rate(%)Ψ** |
| --- | --- | --- | --- | --- | --- |
| 8 | Iavarone M,2011 | Italy | - | 9.2(95%CI:6.4-12.0)* | 7.26 |

* The 95% confidence interval was used as the range for sensitivity analysis.

ΨCalculated from the TTP using the DEALE method as described above.
