# Supplementary material for: Sorafenib versus Transarterial chemoembolization for advanced-stage hepatocellular carcinoma: a cost-effectiveness analysis
Source: BMC Cancer. 2018 Apr 5;18:392. doi: 10.1186/s12885-018-4308-7 (PMC5887167; doi:10.1186/s12885-018-4308-7)
Supplement: Supplementary file 8 — Table S7. References used to derive monthly mortality of patients with compensated cirrhosis and progressive HCC after TACE. (DOCX 13 kb) [file 12885_2018_4308_MOESM8_ESM.docx]

**Supplementary Table 7. References used to derive monthly mortality of patients with compensated cirrhosis and progressive HCC after TACE★**

| **Reference** | **Author, publication year** | **Centre** | **Sample**  **size** | **Median survival**  **(months)** | **Monthly**  **rate(%)Ψ** |
| --- | --- | --- | --- | --- | --- |
| 36 | Cabibbo G,2012 | Italy | 124 | 6.9 | 9.56 |
| 36 | Cabibbo G,2012 | Italy | 102 | 1.8 | 31.96 |
| 37 | Giannini EG,2015 | Italy | 138 | 7 | 9.43 |
| 37 | Giannini EG,2015 | Italy | 210 | 6 | 10.91 |
| 41 | Llovet JM,1999 | Spain | 54 | 5.4 | 12.04 |

★We assumed that the mortality of patients in this state was equivalent to the median of that of patients with BCLC stage C or BCLC stage D HCC. More description was reported in the Materials and Methods.

ΨCalculated from the median survival using the DEALE method as described above.
