# Supplementary material for: Sorafenib versus Transarterial chemoembolization for advanced-stage hepatocellular carcinoma: a cost-effectiveness analysis
Source: BMC Cancer. 2018 Apr 5;18:392. doi: 10.1186/s12885-018-4308-7 (PMC5887167; doi:10.1186/s12885-018-4308-7)
Supplement: Supplementary file 9 — Table S8. References used to derive monthly mortality of patients with compensated cirrhosis and progressive HCC taking sorafenib in full dose or adjusted dose. (DOCX 12 kb) [file 12885_2018_4308_MOESM9_ESM.docx]

**Supplementary Table 8. References used to derive monthly mortality of patients with compensated cirrhosis and progressive HCC taking sorafenib in full dose or adjusted dose**

| **Reference** | **Author, publication year** | **Centre** | **Sample**  **size** | **Median survival**  **(months)** | **Monthly**  **rate(%)Ψ** |
| --- | --- | --- | --- | --- | --- |
| 46 | Lee IC,2015 | China | 63 | 4.6 (95%CI:2.7-6.6) | 13.99  (95%CI:9.97-22.64) |
| 47 | Iavarone M,2015 | Italy | 123 | 4.6 | 13.99 |

ΨCalculated from the median survival using the DEALE method as described above.
