# Supplementary figures and images for: Sorafenib versus Transarterial chemoembolization for advanced-stage hepatocellular carcinoma: a cost-effectiveness analysis
Source: BMC Cancer. 2018 Apr 5;18:392. doi: 10.1186/s12885-018-4308-7 (PMC5887167; doi:10.1186/s12885-018-4308-7)

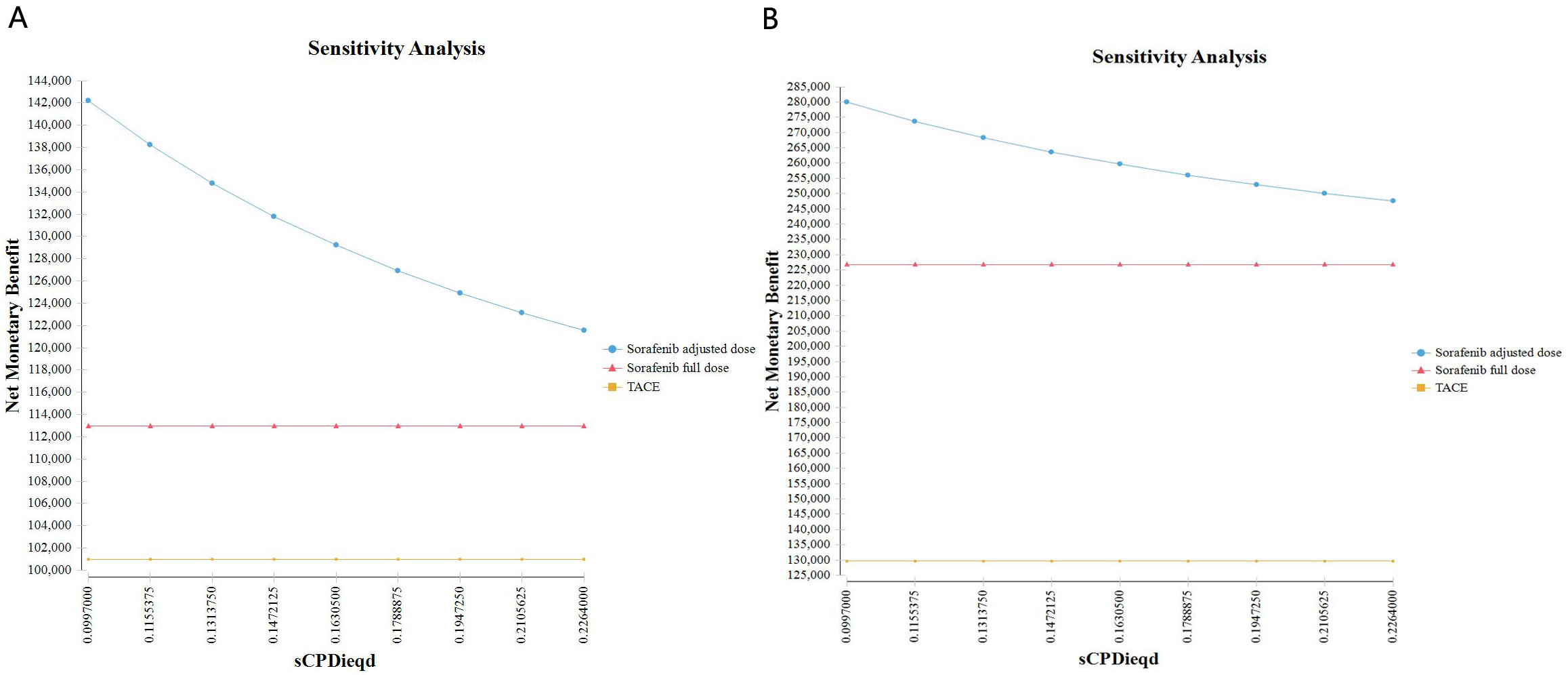

Supplement: Supplementary file 11 — Figure S1. One-way sensitivity analysis of sCPDieqd for the NMB in China (A) and the USA (B). The axis of abscissa represented the range of variable showed in Table 1. The Y-axis represented the value of NMB. The strategy with a higher NMB indicates a cost-effective strategy within the preset WTP. As the rate of sCPDieqd increased, the cost-effectiveness of dose-adjusted sorafenib treatment reduced. sCPDieqd: the mortality of compensated cirrhotic patients with progression taking sorafenib in adjusted dose. (TIFF 670 kb) [file 12885_2018_4308_MOESM11_ESM.tif]

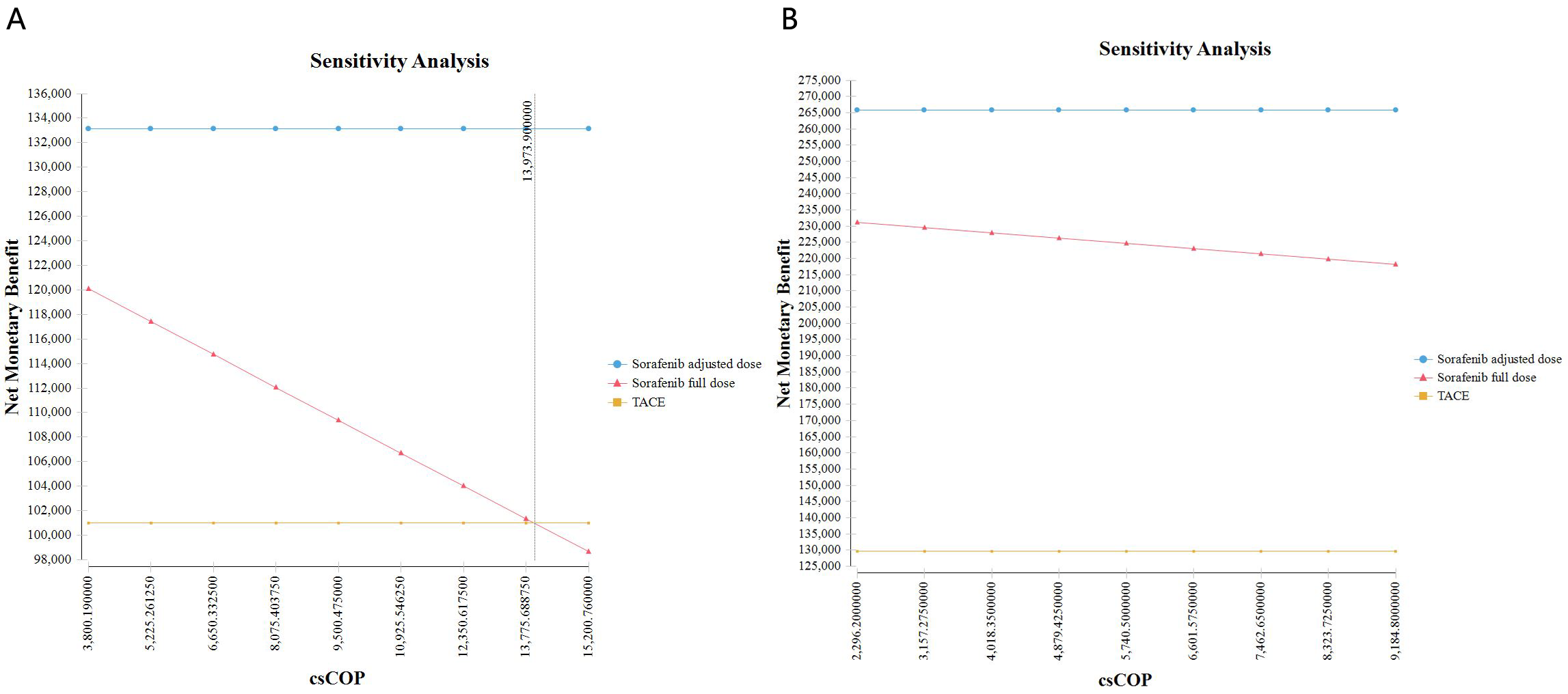

Supplement: Supplementary file 12 — Figure S2. One-way sensitivity analysis of csCOP for the NMB in China (A) and the USA (B). The axis of abscissa represented the range of variable showed in Table 2. The Y-axis represented the value of NMB. A strategy with a higher NMB indicates a cost-effective strategy within the preset WTP. As the value of csCOP increased, the cost-effectiveness of full-dose sorafenib treatment reduced. csCOP: the cost of sorafenib in compensated cirrhotic patients without progression taking sorafenib in full-dose. (TIFF 694 kb) [file 12885_2018_4308_MOESM12_ESM.tif]
